# Broadband Optical Properties of Atomically Thin PtS_2_ and PtSe_2_

**DOI:** 10.3390/nano11123269

**Published:** 2021-12-01

**Authors:** Georgy A. Ermolaev, Kirill V. Voronin, Mikhail K. Tatmyshevskiy, Arslan B. Mazitov, Aleksandr S. Slavich, Dmitry I. Yakubovsky, Andrey P. Tselin, Mikhail S. Mironov, Roman I. Romanov, Andrey M. Markeev, Ivan A. Kruglov, Sergey M. Novikov, Andrey A. Vyshnevyy, Aleksey V. Arsenin, Valentyn S. Volkov

**Affiliations:** 1 Center for Photonics and 2D Materials, Moscow Institute of Physics and Technology, 9 Institutsky Lane, 141700 Dolgoprudny, Russia; georgiy.ermolayev@phystech.edu (G.A.E.); voronin.kv@phystech.edu (K.V.V.); mikhail.tatmyshevskiy@phystech.edu (M.K.T.); arslan.mazitov@phystech.edu (A.B.M.); slavich.as@phystech.edu (A.S.S.); dmitrii.yakubovskii@phystech.edu (D.I.Y.); tselin.ap@phystech.edu (A.P.T.); mironov.ms@phystech.edu (M.S.M.); markeev.am@phystech.edu (A.M.M.); kruglov.ia@mipt.ru (I.A.K.); novikov.s@mipt.ru (S.M.N.); andrey.vyshnevyy@phystech.edu (A.A.V.); arsenin.av@mipt.ru (A.V.A.); 2 Dukhov Research Institute of Automatics (VNIIA), 22 Suschevskaya St., 127055 Moscow, Russia; 3 Moscow Engineering Physics Institute, National Research Nuclear University MEPhI, 31 Kashirskoe Sh., 115409 Moscow, Russia; limpo2003@mail.ru; 4 GrapheneTek, Skolkovo Innovation Center, 143026 Moscow, Russia

**Keywords:** transition metal dichalcogenides, two-dimensional materials, optical constants, dielectric properties, refractive index, nano-photonics, spectroscopic ellipsometry

## Abstract

Noble transition metal dichalcogenides (TMDCs) such as PtS_2_ and PtSe_2_ show significant potential in a wide range of optoelectronic and photonic applications. Noble TMDCs, unlike standard TMDCs such as MoS_2_ and WS_2_, operate in the ultrawide spectral range from ultraviolet to mid-infrared wavelengths; however, their properties remain largely unexplored. Here, we measured the broadband (245–3300 nm) optical constants of ultrathin PtS_2_ and PtSe_2_ films to eliminate this gap and provide a foundation for optoelectronic device simulation. We discovered their broadband absorption and high refractive index both theoretically and experimentally. Based on first-principle calculations, we also predicted their giant out-of-plane optical anisotropy for monocrystals. As a practical illustration of the obtained optical properties, we demonstrated surface plasmon resonance biosensors with PtS_2_ or PtSe_2_ functional layers, which dramatically improves sensor sensitivity by 60 and 30%, respectively.

## 1. Introduction

During the last decade, atomically thin transition metal dichalcogenides (TMDCs) have revolutionized optoelectronics [1,2,3,4,5] thanks to their unique optical and electronic properties, including thickness-dependent bandgap [6], high carrier mobility [7], giant anisotropy [8], high refractive index [9,10], strain-dependent properties [11] and strongly bound excitons [12]. The most well-known materials with these phenomena are the group-VI TMDCs with general structure MX_2_, where M = Mo or W and X = S, Se, or Te [13]. However, despite their enormous potential and tremendous results, they have two significant constraints. First, their bandgap ranges from 1 to 2 eV [14], making group-VI TMDCs rather limited to visible range applications. Second, group-VI TMDCs have low environmental stability [15], which significantly reduces their application possibilities. These problems motivated an intensive search for stable layered materials with a bandgap in the infrared range.

As a result, approximately 5000 potentially useful two-dimensional materials have recently been found [16]. Among them, group-X noble TMDCs (with general structure MX_2_, where M = Pt or Pd and X = S, Se, or Te) stands out, owing to their widely tunable bandgap from visible (for monolayers) to mid-infrared (for few-layers) spectral intervals [17], high electron mobility [18] and remarkable air and liquid stability [19,20]. Broadband photodetectors [21], lasing [22], field-effect transistors [23], label-free sensors [20,24], holography [25], and ultrathin lenses [26], for example, have previously proved the advantages of atomically thin films (about 5 nm) of group-X TMDCs for optoelectronics. With such a wide spectrum of optoelectronic applications, precise knowledge of group-X TMDCs optical properties is of paramount importance. The reported experimental works focused on Raman fingerprints [27,28], absorbance [18,29], and photoconductivity [30,31] of group-X TMDCs. However, there are limited reports [25,32,33,34] on their optical constants (refractive index *n* and extinction coefficient *k*), which are crucial for predicting the performance of optoelectronic devices. Furthermore, these works [25,32,33,34] provide data only for a narrow spectral range required for their specific task. Therefore, a determination of the broadband dielectric function for group-X TMDCs is in high demand.

This work focuses on the optical properties of atomically thin PtS_2_ and PtSe_2_, which are typical representatives of group-X TMDCs. Through spectroscopic ellipsometry measurements, we accurately retrieved their broadband optical constants from ultraviolet to mid-infrared wavelengths (from 245 to 3300 nm). For all of the measured wavelengths, we discovered that PtS_2_ and PtSe_2_ exhibit non-zero extinction coefficients (*k* > 0), which explains recent advances of PtS_2_ and PtSe_2_ in photodetection [21] for these spectral intervals. Additionally, our findings reveal a high refractive index (*n* ~ 4) of these materials, which makes them perfect candidates for all-dielectric nano-photonics [35,36,37].

## 2. Materials and Methods

### 2.1. Materials

Full area coverage PtS_2_ and PtSe_2_ multilayers were purchased from 2d Semiconductors, Inc. (2d Semiconductors Inc., Scottsdale, AZ, USA). The samples were grown on c-cut sapphire substrates by chemical vapor deposition (CVD) using the highest purity (6N) gases (N_2_/H_2_) and precursors (S powder and Pt films) in semiconductor-grade facilities with subsequent water-assisted transfer on a 300 nm SiO_2_/Si substrate.

### 2.2. Raman Characterization

The experimental setup used for Raman measurements was a Horiba LabRAM HR Evolution confocal scanning Raman microscope (Horiba Ltd., Kyoto, Japan). All measurements were carried out using linearly polarized excitation at wavelength 632.8 nm; 1800 lines/mm diffraction grating, and × 100 objective (N.A. = 0.90), whereas we used unpolarized detection to have a significant signal-to-noise ratio. The spot size was approximately 0.43 µm. The Raman spectra were recorded with 0.75 mW and an integration time of 10 s at each point. The statistics were collected with 15 points for each sample, and the observed variation of the intensity for the spectra was less than 5%.

### 2.3. XPS Characterization

The chemical state of the elements in the film was analyzed by X-ray photoelectron spectroscopy (XPS) in the Theta Probe tool (Thermo Scientific K-Alpha, Waltham, MA, USA) under ultrahigh vacuum conditions (base pressure < 10^−9^ mBar) with a monochromatic Al-K_α_ X-ray source (1486.6 eV). Photoelectron spectra were acquired using fixed analyzer transmission (FAT) mode with 50 eV pass energy. The spectrometer energy scale was calibrated using C1s line position at 284.5 eV.

### 2.4. Atomic Force Microscopy

The thickness and surface morphology of PtS_2_ and PtSe_2_ films were accurately characterized by an atomic force microscope (NT-MDT N’tegra tool, Moscow, Russia) using AFM in peak-force mode under ambient conditions. AFM measurements were carried out using ETALON, HA_NC silicon tips from TipsNano (TipsNano, Tallin, Estonia) with a spring constant of 3.5 N/m, a head curvature radius < 10 nm and a resonant frequency of 140 kHz. Images of PtS_2_ and PtSe_2_ surfaces were taken over a 3 × 3 µm^2^ area with a scan rate of 0.2 Hz; after that, data were analyzed by Gwyddion software.

### 2.5. Optical Visualization

The surface images (2400 × 2400 pixels) of PtS_2_ and PtSe_2_ were captured by an optical microscope (Nikon LV150L, Tokyo, Japan) with a digital camera DS-Fi3.

### 2.6. Scanning Electron Microscopy

A scanning electron microscope JEOL JSM-7001F (JEOL Ltd., Tokyo, Japan) accompanied by a Schottky emitter in secondary electron imaging mode with a voltage of 30 keV and current of 67 µA, and a working distance of approximately 6.3 mm, was used to study surface features and homogeneity of PtS_2_ and PtSe_2_ films surfaces in detail within different areas using a 1960 × 1280 pixel scan.

### 2.7. X-ray Diffraction

An X-ray powder diffractometer (XRD, Thermo ARL X’TERA, Waltham, MA, USA) equipped with Cu K_α_ radiation λ = 0154 nm was used to characterize the crystalline structure and phase of PtS_2_ and PtSe_2_ films. The XRD pattern was taken at ambient conditions by 2*θ*-scan over the range of 20–75° with a step of 0.05° and accumulation time of 2 s.

### 2.8. Reflectance Measurements

The spectroscopic reflection analysis was performed in the 400–975 nm spectral range on a Biolam M-1 microscope (LOMO, Saint-Petersburg, Russia) equipped with a 24 V, 100 W halogen light source and a QE65000 fiber-coupled grating spectrometer (Ocean Optics). The reflected light was collected in a backscattering configuration using an objective with magnification 100× (NA = 0.80). The experimental data represent the reflection ratio *R*_str_/*R*_ref_, where *R*_str_ is the reflection measured from the structures with films and *R*_ref_ is the reference from a silver mirror NT64−114 (Edmund Optics, Barrington, NJ, USA) that exhibits an average reflection of 99% between 350 and 1100 nm of light wavelength.

### 2.9. Ellipsometry Characterization

We used a variable-angle spectroscopic ellipsometer (VASE, J.A. Woollam Co., Lincoln, NE, USA) with a single chamber monochromator with two gratings: 1200 g/mm for visible light (245–1040 nm) with 4.6 nm bandwidth and 600 g/mm for the infrared interval (1040–3300 nm) with 9.2 nm bandwidth. Spectroscopic ellipsometry was conducted over a wide wavelength range (from 245 to 3300 nm in steps of 1 nm) and multiple angles of incidence in the range of 30° to 80° with a step size of 5°.

### 2.10. Mueller Matrix Measurements

To investigate the in-plane anisotropic response of PtS_2_ and PtSe_2_, we measured 11 elements of the Mueller matrix (m_12_, m_13_, m_14_, m_21_, m_22_, m_23_, m_24_, m_31_, m_32_, m_33_, m_34_) on an Accurion nanofilm_ep4 ellipsometer (Accurion GmbH, Goettingen, Germany) at 532 nm and 50° incident angle in rotation compensator mode.

### 2.11. First-Principle Calculations

The optical properties of PtS_2_ and PtSe_2_ were calculated using density functional theory (DFT) within the generalized gradient approximation [38] (Perdew–Burke–Ernzerhof functional) and the projector-augmented wave method [39] as implemented in the Vienna Ab Initio Simulation Package. The unit cell parameters of PtS_2_ were *a* = *b* = 0.3537 nm, *c* = 0.5019 nm, *α* = *β* = 90°, *γ* = 120°, and *a* = *b* = 0.3731 nm, *c* = 0.5072 nm, *α* = *β* = 90°, *γ* = 120° for PtSe_2_ [40]. A two-step approach was used: First, the atomic positions of PtS_2_ and PtSe_2_ were relaxed until the interatomic forces were less than 10^−3^ eV/Å, and a one-electron basis set was obtained from standard DFT calculations. Second, the real and imaginary parts of the frequency-dependent dielectric function were calculated using the GW approximation [41]. In addition, the spin–orbit interaction was included in the calculation to account for relativistic corrections to the dielectric function. The plane-wave kinetic energy cutoff was set to 700 eV, and the Γ-centered 15 × 11 × 11 k-points mesh was used to sample the first Brillouin zone.

## 3. Results and Discussion

### 3.1. Samples Characterization

**Figure 1:**
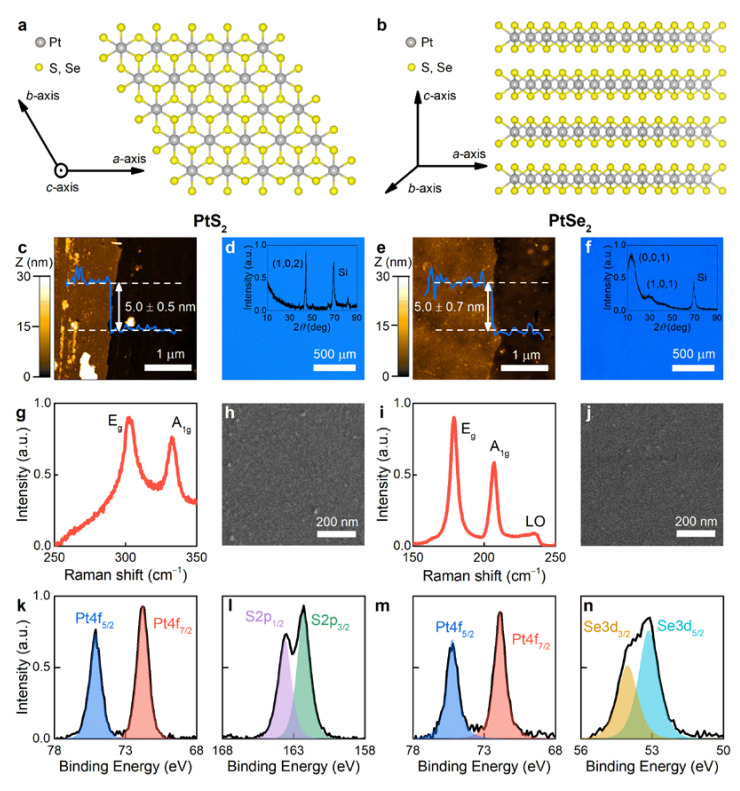
Characterization of PtS_2_ and PtSe_2_ films. Crystal structure of PtS_2_ and PtSe_2_ from different views along (**a**) (001) and (**b**) (210) directions. AFM topography mappings and cross-sectional profiles of the edge of (**c**) PtS_2_ and (**e**) PtSe_2_. Optical images of (**d**) PtS_2_ and (**f**) PtSe_2_ on top of 300 nm SiO_2_/Si substrate. The insets show XRD diffraction patterns for PtS_2_ and PtSe_2_, respectively. Raman spectra at excitation wavelength λ = 632.8 nm of (**g**) PtS_2_ and (**i**) PtSe_2_ show characteristic Raman modes E_g_ and A_1g_. Note that PtSe_2_ also has an additional peak labelled LO (longitudinal optical) resulting from the overlap between infrared active modes E_u_ and A_2u_ [27]. SEM images of (**h**) PtS_2_ and (**j**) PtSe_2_. XPS spectra of (**k**,**l**) PtS_2_ and (**m**,**n**) PtSe_2_.

Atomically thin PtS_2_ and PtSe_2_ were prepared by chemical vapor deposition (CVD) on c-cut sapphire with subsequent water-assisted transfer on a 300 nm SiO_2_/Si substrate [42] to facilitate spectroscopic ellipsometry studies of optical constants, owing to interference in the thick silicon oxide. PtS_2_ and PtSe_2_ grow in the thermodynamically favored 1T-phase, as illustrated in Figure 1a,b, unlike group-VI TMDCs [43]. As shown in Figure 1c and e, the CVD-grown PtS_2_ and PtSe_2_ have a thickness of 5 nm determined by atomic force microscopy (AFM). Therefore, our films have ten layers, since the interlayer distance in PtS_2_ and PtSe_2_ is 0.5 nm [27]. Raman spectroscopy in Figure 1g,i of the obtained films reveals pronounced peaks inherent to PtS_2_ and PtSe_2_ Raman modes E_g_ and A_1g_, corresponding to in-plane and out-of-plane vibrations of chalcogen atoms (S, Se), respectively [43]. Indeed, their position (E_g_ ~ 300 cm^−1^ and A_1g_ ~ 335 cm^−1^ for PtS_2_; E_g_ ~ 175 cm^−1^ and A_1g_ ~ 205 cm^−1^ for PtS_2_) corresponds to few-layer PtS_2_ and PtSe_2_ [27,28] in agreement with AFM measurements. The Raman spectra do not contain photoluminescence responses in agreement with previous reports [27,28] on Raman study of PtS_2_ and PtSe_2_ at the 632.8 nm excitation wavelength. Moreover, our samples uniformly cover the substrate as confirmed by optical and scanning electron microscopy (SEM) images for PtS_2_ and PtSe_2_ in Figure 1d,h. Therefore, our samples are uniform at different scales, which is understood from the uniform color and contrast in optical and SEM images, respectively. One may notice small features of about 10 nm seen in the SEM images (Figure 1h,j), which are leftovers of the transfer process from sapphire to SiO_2_/Si substrate. Nevertheless, these leftovers cover less than 5% of the surface and, hence, have a negligible effect on the resulting optical constants of PtS_2_ and PtSe_2_ studied here [44]. Additionally, X-ray photoemission spectroscopy (XPS) in Figure 1k–n shows Pt 4f, S 2p, and Se 3d spectra associated with PtS_2_ and PtSe_2_ [27,28]. Finally, the crystallinity of the synthesized PtS_2_ and PtSe_2_ films was shown by measuring the X-ray diffraction (XRD) spectra displayed in the inset of Figure 1d,f.

### 3.2. Dielectric Response Analysis

**Figure 2:**
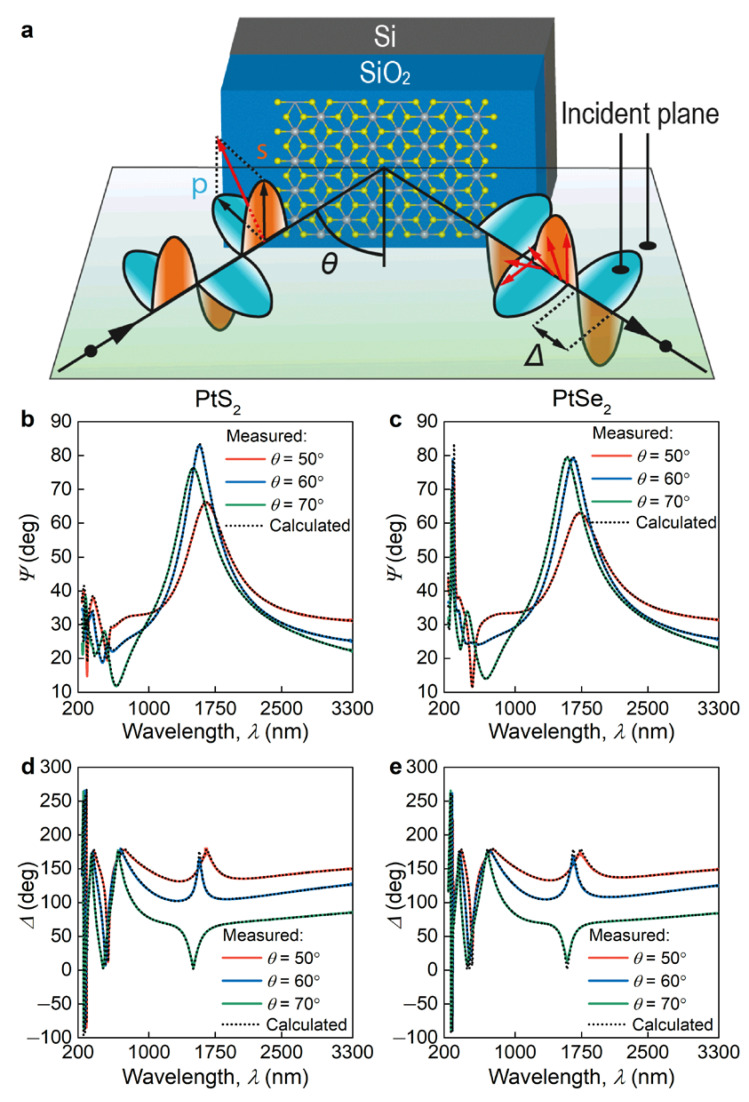
Ellipsometry of PtS_2_ and PtSe_2_. (**a**) Schematics of the spectroscopic ellipsometry experimental configuration used to determine PtS_2_ and PtSe_2_ optical constants. (**b**–**e**) Plots of the exemplified measured (solid lines) and calculated (dashed lines) ellipsometric spectra of Ψ and Δ of PtS_2_ and PtSe_2_ on SiO_2_/Si substrate.

**Figure A1:**
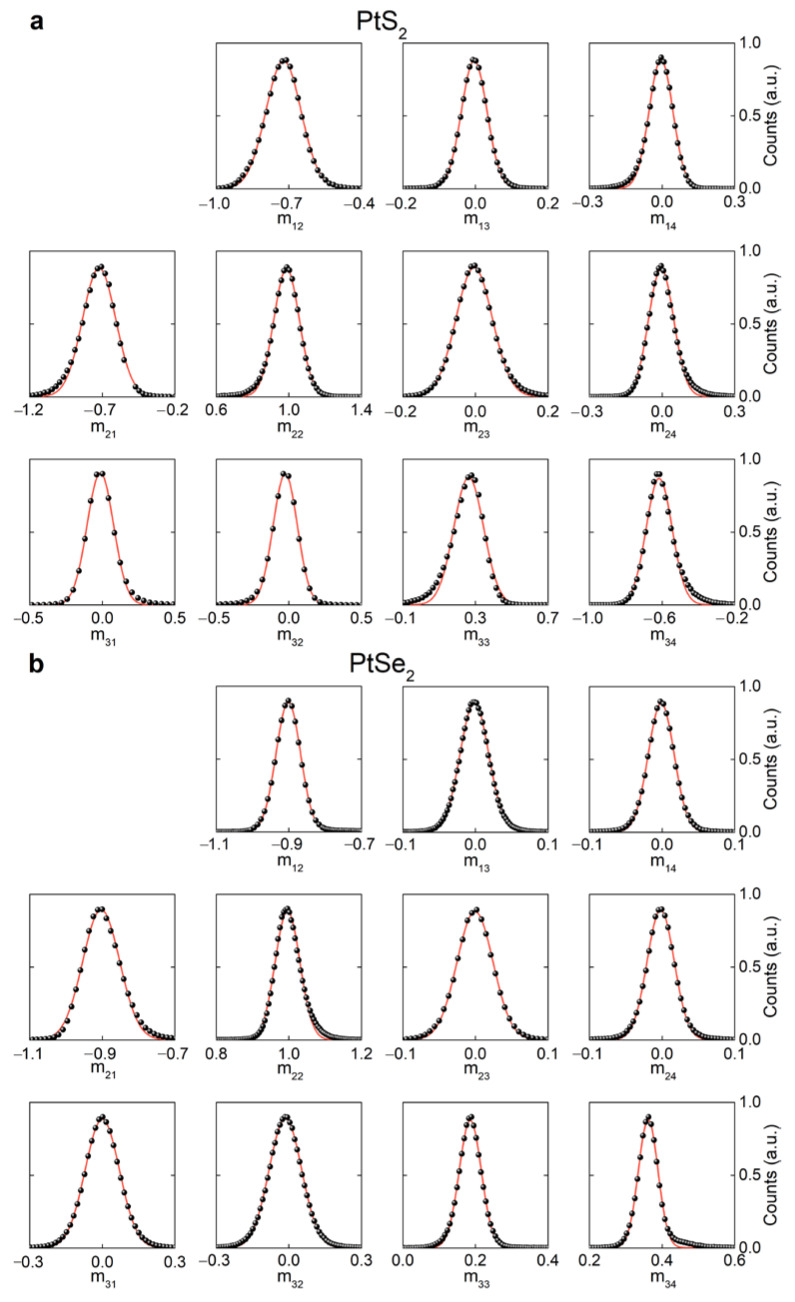
Distribution of Mueller matrix values. (**a**) PtS_2_ and (**b**) PtSe_2_. Mueller Matrix values recorded at 50° and λ = 532 nm. Relative frequency of Mueller matrix values follows Gaussian distribution (red line) with zero average for non-diagonal blocks (m_13_, m_14_, m_23_, m_24_, m_31_, m_32_), thus validating isotropic in-plane response.

**Figure A2:**
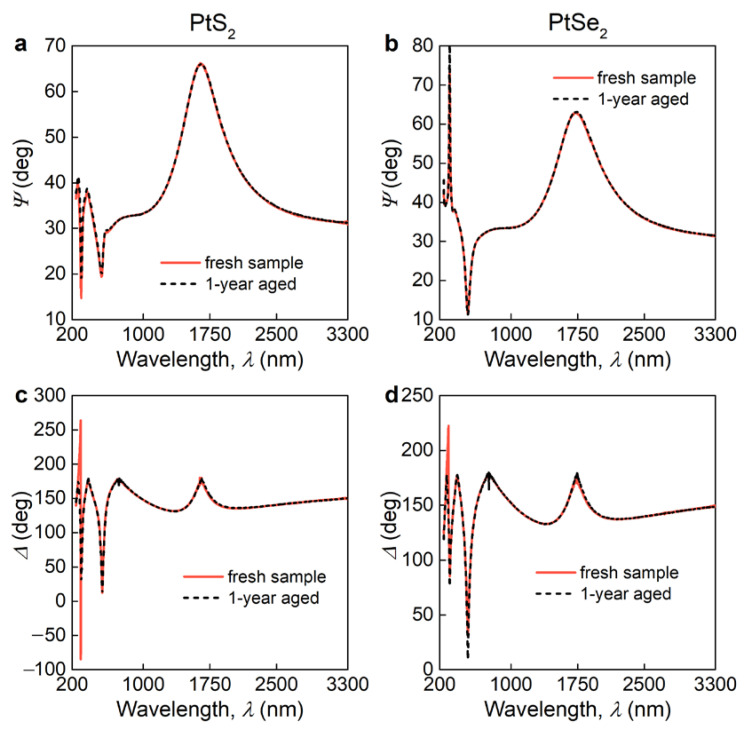
Stability of PtS_2_ and PtSe_2_ optical response. Plots of (**a**,**b**) Ψ and (**c**,**d**) Δ for freshly synthesized (solid red line) and one year aged in air (dashed black line) PtS_2_ and PtSe_2_ on SiO_2_/Si substrate.

To properly quantify broadband optical properties of atomically thin PtS_2_ and PtSe_2_, we performed spectroscopic ellipsometry (SE) measurements at multiple incident angles (30–80° in 5° steps) and wavelengths (245–3300 nm in 1 nm steps). The experimental scheme of SE setup is displayed in Figure 2a. SE measures the change in polarization upon reflection in terms of Ψ and Δ (Figure 2b–e), which depends on the optical constants of the investigated sample. Hence, we need to provide an optical model to retrieve the dielectric function of PtS_2_ and PtSe_2_. First, we checked the in-plane anisotropy of our samples using the Mueller matrix method [20], in which non-zero non-diagonal elements account for in-plane optical anisotropy. In our case, zero non-diagonal elements of the Mueller matrix (Figure A1) clearly indicate the isotropic optical response of PtS_2_ and PtSe_2_ in agreement with the previous study [32]. In addition, we recorded the Ψ and Δ spectra for one-year aged samples (Figure A2), which reproduced the data in Figure 2b–e, thereby confirming the stability of PtS_2_ and PtSe_2_.

**Figure 3:**
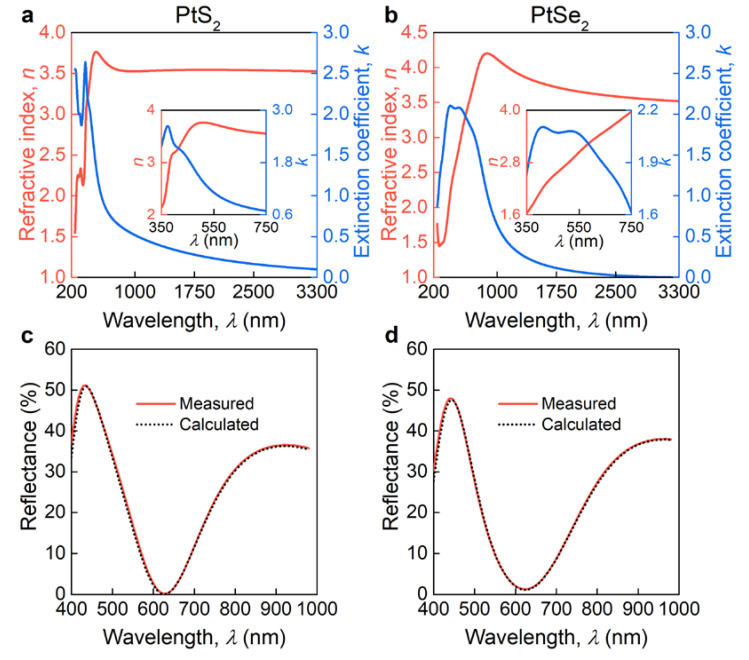
Optical properties of PtS_2_ and PtSe_2_. Optical constants of (**a**) PtS_2_ and (**b**) PtSe_2_. The insets show PtS_2_ and PtSe_2_ optical constants in the visible range. Measured and calculated reflectance spectra for (**c**) PtS_2_ and (**d**) PtSe_2_ on SiO_2_/Si substrate.

**Figure A3:**
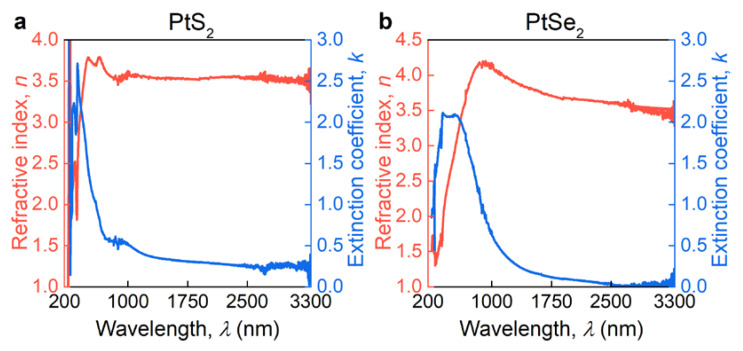
Point-by-point fitting of Ψ and Δ. Direct inversion of Ψ and Δ from Figure 2 in optical constants for (**a**) PtS_2_ and (**b**) PtSe_2_.

**Figure A4:**
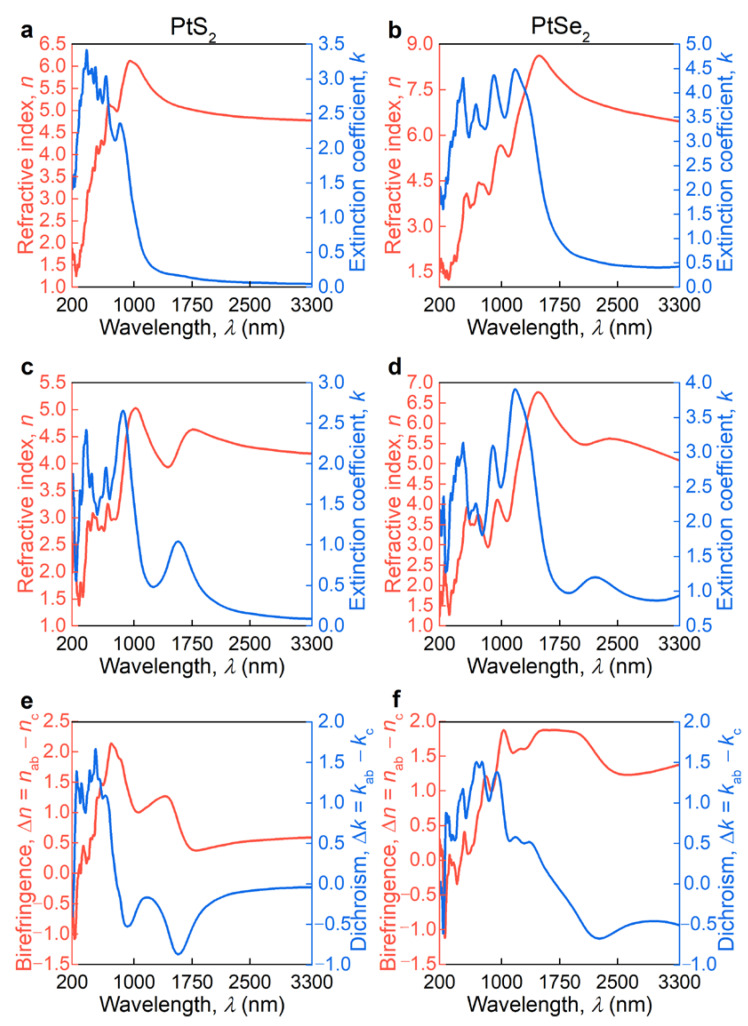
First-principle calculations of PtS_2_ and PtSe_2_ optical constants. (**a**,**b**) In-plane optical constants and (**c**,**d**) out-of-plane optical constants. (**e**,**f**) Out-of-plane anisotropy.

For the optical model, we used the thickness determined from AFM (Figure 1c,e), which allowed us to fix the thickness during the fitting procedure. Note that some authors fit the thickness of thin films (<10 nm) and optical constants at the same time. However, such an approach usually leads to incorrect thickness and optical constants results because of their high correlation [45]. We also used point-by-point inversion [44] to obtain the initial approximation of PtS_2_ and PtSe_2_ optical constants (Figure A3). In this approach, for each wavelength, refractive index n and extinction coefficient k are varied to achieve the best match with experimental spectra. Despite its effectiveness and ease of use, this method results in noisy data and sometimes unphysical values [45]. In contrast, the oscillator approach leads to smooth and the Kramers–Kronig consistent dielectric function [46]. As a result, in the next step, we used the Tauc–Lorentz oscillator model, which is commonly used for optical modeling of TMDCs [46,47,48]:

(1) $$\varepsilon_{2}=\{\begin{array}{cc} \frac{1}{E}\cdot \frac{AE_{0}C{\left(E-E_{g}\right)}^{2}}{{\left(E^{2}-E_{0}^{2}\right)}^{2}+C^{2}E^{2}} & for\;E>E_{g}\\ 0 & for\;E<E_{g} \end{array}$$

where E is the photon energy, A is the oscillator strength, C is the oscillator broadening, $E_{g}$ is the optical bandgap, and $E_{0}$ is the oscillator central energy, while the real part $\varepsilon_{1}$ of the dielectric function is derived from Kramers–Kronig integration plus $\varepsilon_{\infty }$ to account for high energy electronic transitions. After fitting the oscillator parameters (Table 1 and Table 2), we obtained the final PtS_2_ and PtSe_2_ optical constants depicted in Figure 3a,b, which yield the perfect agreement between calculated and experimental Ψ and Δ (Figure 2b,e). The resulting oscillator parameters are collected in Table 1 and Table 2. Further, to confirm our optical constants, we recorded the reflectance spectra (Figure 3c,d) [49] and compared them with the transfer matrix calculations [50] based on the dielectric function from Figure 3a,b. Figure 3c,d show the perfect match between calculated and experimental spectra, which additionally verifies our optical constants. It is worth noting that the oscillations in the reflectance spectra (Figure 3c,d) originate from thin film interference in the SiO_2_ layer [51], which enhances the light–matter interaction with our samples. Of immediate interest is also the refractive index and extinction coefficient values of PtS_2_ and PtSe_2_: both materials have *k* > 0 in the entire spectral range and high refractive index *n* ~ 4. In contrast, group-VI TMDCs such as MoS_2_ and WS_2_ have zero extinction coefficient, but a similar refractive index of about 4 in the infrared range [9,46]. We also retrieved the optical constants from the first-principle calculations under the assumption of perfect crystallinity (see Methods and Figure A4a,d). Although the theoretical values deviate from experimental values due to approximation methods and the polycrystalline structure of CVD-grown films, first-principle calculations capture the major optical features of PtS_2_ and PtSe_2_: broadband absorption and strong dielectric response. Furthermore, theory predicts a giant out-of-plane optical anisotropy (Figure A4e,f), making PtS_2_ and PtSe_2_ ideal candidates for recently emerging anisotropic nano-photonics [8]. Therefore, PtS_2_ and PtSe_2_ are particularly promising for optoelectronics and nano-photonics, since their out-of-plane anisotropy provides an extra degree of freedom, non-zero *k* yields efficient light-harvesting, and large *n* leads to efficient concentration of electromagnetic energy.

**Figure 4:**
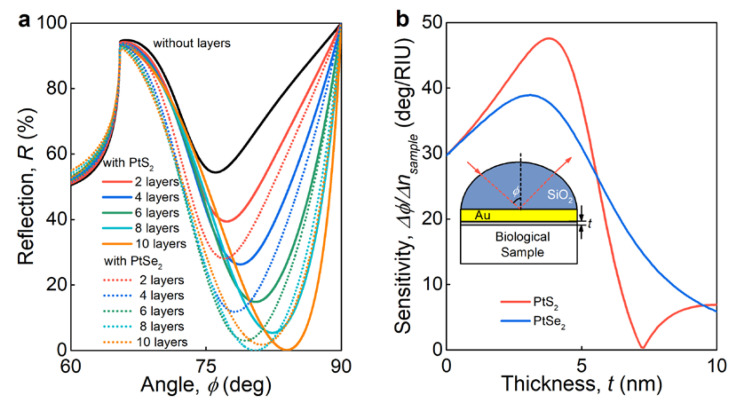
Surface plasmon resonance (SPR) biosensor based on PtS_2_ and PtSe_2_**.** (**a**) The reflectance spectra of SPR sensor for different layer numbers of PtS_2_ and PtSe_2_. (**b**) The dependence of the SPR sensor sensitivity on PtS_2_ and PtSe_2_ thickness. The inset is a schematic configuration of an SPR sensor. Calculations performed at 635 nm wavelength.

To highlight PtS_2_ and PtSe_2_ usage in photonic devices, we considered a label-free biosensor based on surface plasmon resonance (SPR) in the Kretschmann [52] configuration, where a thin gold film (25 nm) covers a silicon oxide prism with PtS_2_ or PtSe_2_ as functional layers. In this scheme, the change in refractive index of a biological sample is detected, which involves monitoring the resonant reflection shift of the minimum (Figure 4a). As seen in Figure 4, PtS_2_ and PtSe_2_ films considerably enhance the structure sensitivity by 60 and 30% (Figure 3a,b), respectively, thanks to their high refractive index, which enhances SPR near-field interaction with the biological sample [53]. Conversely, the extinction coefficient leads to absorption of surface plasmons [54]. These two factors determine the optimal thickness of the functional layer (PtS_2_ or PtSe_2_) of about 4 nm with maximum refractive index sensitivity, as seen in Figure 4b. As a result, the superior optical response of PtS_2_ and PtSe_2_ improves device performance and, hence, could be used in numerous applications in optoelectronics and photonics.

## 4. Conclusions

In conclusion, we report broadband (245–3300 nm) optical properties for atomically thin PtS_2_ and PtSe_2_ films. We unveiled their ultrawide absorption and strong dielectric response, explaining the recent technological advancement of PtS_2_ and PtSe_2_-based optoelectronic devices. Moreover, we confirmed our PtS_2_ and PtSe_2_ optical constants both theoretically (first-principle calculations) and experimentally (reflectance measurements). Finally, we demonstrated that PtS_2_ and PtSe_2_ could serve as a functional layer in biosensors based on surface plasmon resonance. Altogether, these findings provide a foundation for PtS_2_ and PtSe_2_ optoelectronic and photonic devices, including label-free sensors [24], ultrasensitive broadband photodetectors [21], and ultrathin lenses [26].

## Figures and Tables

**Table 1:** Tauc–Lorentz parameters of the oscillators (excitons) with $\varepsilon_{\infty }$ = 1.766 and $E_{g}$ = 0.137 eV used to describe dielectric function of PtS_2_. Tabulated optical constants are collected in Table A1.

Oscillator	A (eV)	C (eV)	*E*_0_ (eV)
#1	13.177	6.154	2.595
#2	13.274	1.183	2.879
#3	1.879	0.328	3.268
#4	0.905	0.440	4.000
#5	11.197	1.338	4.759

**Table 2:** Tauc–Lorentz parameters of the oscillators (excitons) with $\varepsilon_{\infty }$ = 1.766 and $E_{g}$ = 0.349 eV used to describe dielectric function of PtSe_2_. Tabulated optical constants are collected in Table A1.

Oscillator	A (eV)	C (eV)	*E*_0_ (eV)
#1	8.177	0.734	1.654
#2	14.917	1.307	2.200
#3	10.018	1.469	3.049
#4	2.325	1.399	4.359
#5	6.608	0.530	5.782

## Data Availability

The data presented in this study are available upon reasonable request from the corresponding author.

## Appendix A

**Table A1:** Tabulated optical constants for PtS_2_ and PtSe_2_ films from Figure 3a,b.

	PtS_2_		PtSe_2_	
*λ* (nm)	*n*	*k*	*n*	*k*
250	1.7037	2.5497	1.6559	0.8959
300	2.3991	2.0033	1.4828	1.4051
350	2.1384	2.1745	1.6315	1.8340
400	3.1896	2.2133	2.1016	2.0953
450	3.5743	1.8970	2.4795	2.0778
500	3.7603	1.4317	2.7416	2.0768
550	3.7307	1.1122	3.0327	2.0632
600	3.6664	0.9279	3.3009	1.9786
650	3.6139	0.8156	3.5186	1.8786
700	3.5777	0.7403	3.7384	1.7766
750	3.5543	0.6850	3.9639	1.6139
800	3.5398	0.6414	4.1262	1.3813
850	3.5313	0.6050	4.1946	1.1391
900	3.5268	0.5733	4.1961	0.9328
1200	3.5306	0.4338	3.9555	0.3629
1500	3.5400	0.3387	3.7989	0.1871
1800	3.5437	0.2693	3.7062	0.1050
2100	3.5430	0.2171	3.6446	0.0593
2400	3.5397	0.1768	3.6001	0.0319
2700	3.5350	0.1450	3.5663	0.0153
3000	3.5296	0.1196	3.5400	0.0057
3300	3.5238	0.0990	3.5194	0.0011
